# STAndard Reporting of CAries Detection and Diagnostic Studies (STARCARDDS)

**DOI:** 10.1007/s00784-021-04173-3

**Published:** 2021-10-08

**Authors:** Klaus W. Neuhaus, Florin Eggmann, Jan Kühnisch, Svetlana Kapor, Mila Janjic Rankovic, Ina Schüler, Felix Krause, Adrian Lussi, Stavroula Michou, Kim Ekstrand, Marie-Charlotte Huysmans

**Affiliations:** 1 Department of Periodontology, Endodontology and Cariology, University Center for Dental Medicine Basel UZB, University of Basel, Mattenstrasse 40, 4058 Basel, CH Switzerland; 2 Department of Dermatology, Inselspital - Bern University Hospital, Bern, Switzerland; 3 Department of Conservative Dentistry and Periodontology, University Hospital, Ludwig-Maximilians University Munich, Munich, Germany; 4 Department of Orthodontics and Dentofacial Orthopedics , University Hospital, Ludwig-Maximilians-Universität München, Munich, Germany; 5 Department of Orthodontics, Section of Preventive and Paediatric Dentistry , University Hospital, Jena, Germany; 6 Department Clinic for Operative Dentistry, Periodontology and Preventive Dentistry, University Hospital RWTH Aachen, Aachen, Germany; 7 Department of Operative Dentistry and Periodontology, Faculty of Dentistry, University Medical Centre, Freiburg, Germany; 8 School of Dental Medicine, University of Bern, Bern, Switzerland; 9 Department of Odontology, University Copenhagen, Copenhagen, Denmark; 10 Department of Dentistry, Radboud University Medical Center, Nijmegen, The Netherlands

**Keywords:** Caries detection, Caries diagnostics, Caries assessment, Methodology, Laboratory studies, Reference standard

## Abstract

**Aim:**

The aim of this paper is to present recommendations from an international workshop which evaluated the methodology and reporting of caries diagnostic studies. As a unique feature, this type of studies is focused on caries lesion detection and assessment, and many of them are carried out in vitro, because of the possibility of histological validation of the whole caries spectrum. This feature is not well covered in the existing reporting STARD guideline within the EQUATOR Network.

**Participants and methods:**

An international working group of 13 cariology researchers was formed. The STARD checklist was reviewed and modified for caries detection and diagnosis purposes, in a three-step process of evaluation, consensual modification, and delivery during three 2-day workshops over 18 months. Special attention was paid to reporting requirements of caries studies that solely focus on reliability.

**Results:**

The STARD checklist was modified in 14/30 items, with an emphasis on issues of sample selection (tooth selection in in vitro studies), blinding, and detailed reporting of results.

**Conclusion:**

Following STARCARDDS (STAndard Reporting of CAries Detection and Diagnostic Studies) is expected to result in complete reporting of study design and methodology in future caries diagnosis and detection experiments both in vivo and in vitro, thus allowing for better comparability of studies and higher quality of systematic reviews.

**Clinical relevance:**

Standardization of caries diagnostic studies leads to a better comparability among future studies, both in vivo and in vitro.

## Introduction

There is a wide range of methods currently available for caries detection and diagnosis [1]. In addition, novel diagnostic approaches are being developed while existing methods undergo refinement as the diagnostic thresholds and cutoffs may change over time to meet the requirements of the prevailing caries management principles that they underpin [2, 3].

Caries detection and diagnostic studies aim to assess the accuracy of a diagnostic method, or combinations thereof, with commonly used statistical measures such as sensitivity and specificity, or the reliability of a diagnostic workup or both. Systematic reviews indicate, however, that the quality of reporting in caries detection and diagnostic studies is frequently low [3–9]. Incomplete and inconsistent reporting can preclude a comprehensive evaluation of study methods and the potential of bias. As a consequence, both critical appraisal and replication of a study may be challenging, if not impossible [10–12]. Moreover, systematic reviews and meta-analyses may be severely hampered by insufficient reporting of data in primary studies [12].

To improve the completeness and transparency of reporting in diagnostic accuracy studies, STAndards for Reporting of Diagnostic Accuracy Studies (STARD), a checklist of essential items that ought to be reported in diagnostic studies, was compiled and published in numerous journals in 2003 [13]. This checklist was later revised and the updated version, including 30 essential items, was published in 2015 [12]. The STARD checklist is a valuable methodological tool to enhance, safeguard, and assess the quality of reporting in diagnostic studies. As such, it provides assistance to authors, editors, reviewers, and the readership of diagnostic studies. The CONSORT (Consolidated Standards of Reporting Trial) statement, like STARD part of the EQUATOR Network, provides guidelines to improve the reporting of randomized, controlled trials [14]. Using the CONSORT checklist is strongly recommended to ensure complete, clear, and transparent reporting of randomized, controlled trials. However, randomized, controlled trials investigating caries detection and diagnostic methods are currently scarce. There is a need for a reporting guideline — applicable regardless of the study design — that assists authors in writing reports of caries detection and diagnostic studies.

STARD is applicable to all types of diagnostic tests [12]. The STARD checklist does not, however, fully address some features that are specific to caries diagnostic studies. For instance, there is a majority of studies concentrating on the accuracy of caries lesion detection and (staging) assessment usually in an in vitro setting, but also in vivo in a selected sample of teeth [3, 5, 7, 8]. The composition of the sample of teeth with regard to the presence and depth of caries lesions can vary greatly [3, 5, 9]. So-called spectrum effects that derive from the specific case mix in a sample have a profound impact on the diagnostic performance of a test [15]. Thus, the applicability of the results may be severely restricted whenever the sample is unrepresentative of the target population [15]. A thorough assessment of any spectrum effects therefore relies on the accurate and complete description of the tooth sample. This requires a particular level of detail in the study report, which is not readily identified by the STARD criteria.

The STARD group has welcomed the development of additional instructions for informative reporting for specific applications [12]. The objective of this study was therefore to identify aspects of reporting that are crucial in caries detection/diagnostic studies and, based on that, to develop a STARD extension for this specific domain. This STARD extension, termed STARCARDDS (short for STAndard Reporting of CAries Detection and Diagnostic Studies), should promote and facilitate the completeness and transparency of reporting of future studies in the field of caries detection and diagnosis, both in vivo and in vitro.

## Methods

An international, 3-member steering committee, including JK, KWN, and IS, was responsible for coordinating the development of the STARD extension STARCARDDS. This team secured funding, identified, and invited potential participants for the development process and organized meetings, both in person and virtual. Under the leadership of the steering committee, an international working group, comprising 13 researchers in cariology, was formed. The participating members were from Europe and were internationally recognized experts in the field of caries detection and diagnostic studies. They needed to consent in a three-step process and needed to be physically available at the group meetings. All authors of the present report were members of the STARCARDDS working group. The working group held three 2-day consensus workshops, taking place in Bern, Switzerland (October 16–17, 2017), Berlin, Germany (September 8–9, 2018), and Frauenwörth, Germany (March 25–26, 2019).

To establish a consensus, underpinned by cariologic evidence, on reporting standards for caries detection and diagnosis studies, the STARCARDDS group adopted a step-wise development approach [16]. This development process comprised three broad phases: (1) evaluation, (2) drafting with discussion and feedback, and (3) delivery.

### Phase 1, evaluation

A systematic review was undertaken to assess the risk of bias in caries detection and diagnosis studies [17, 18]. The quality of reporting of included studies was comprehensively evaluated. Based on the findings of the systematic review, the applicability of the existing reporting guideline, STARD, was assessed and STARD items that may benefit from additional or modified reporting recommendations were identified.

Data of the systematic reviews are reported in detail elsewhere [17, 18]. In brief, within established methodological frameworks, two separate systematic reviews were undertaken: one on occlusal surface caries detection and diagnosis and one on proximal surface caries detection and diagnosis. Studies pertaining to primary teeth or teeth with restorations, secondary caries, or artificially induced caries lesions were excluded.

The systematic review of the literature on *occlusal surface* caries detection and diagnosis included in vitro and in vivo diagnostic studies that tested the diagnostic accuracy and/or reliability/reproducibility of different diagnostic methods for primary caries detection and assessment in human permanent posterior teeth. The following index tests were included in the search: visual examination, conventional and digital bitewing radiography, laser fluorescence measurements, fiber-optic transillumination, and quantitative light-induced fluorescence. Use of a reference standard was a requisite feature of studies to be eligible for inclusion. The systematic review of the literature on occlusal surface caries detection and diagnosis initially identified 140 studies out of a total of 1090 screened records. A total of 103 publications needed to be excluded owing to a high risk of bias or insufficient data reporting. Finally, 29 in vitro and 8 in vivo studies were selected according to a stepwise eligibility assessment.

The systematic review of the literature on *proximal surface* caries detection and diagnosis included in vivo and in vitro caries diagnostic studies that tested the diagnostic performance of the following caries diagnostic methods: visual examination with and without tactile examination, conventional and digital bitewing radiography, laser fluorescence measurement, and fiber-optic transillumination. Only studies assessing primary caries on the proximal surfaces of permanent posterior teeth were considered for inclusion. The actual status of the tooth surface had to be confirmed by a reference standard. In order to be included, at least one of the following outcomes had to be assessed: diagnostic test accuracy or reliability/reproducibility.

In total, out of 851 screened records, 129 studies met the inclusion criteria in the first selection step of the systematic review of the literature on proximal surface caries detection. When additionally considering those studies with a low/moderate risk of bias, the number of includable studies decreased to 43, of which 7 studies had to be excluded owing to low quality of data reporting. Finally, 31 laboratory studies and 5 clinical studies were included in the meta-analysis.

### Phase 2, drafting with discussion and feedback

Supplementary recommendations, specific for caries detection and diagnosis studies, were drafted and discussed. A consensual draft version of STARCARDDS checklist was decided upon on March 26, 2019. After the last face-to-face meeting, the working group produced a final draft, collecting feedback and holding further discussions throughout the drafting process.

### Phase 3, delivery

Publication in an international peer reviewed journal.

## Results

In result of this consensus process, the STARCARDDS checklist was developed which can be taken from Table 1.

**Table 1:**
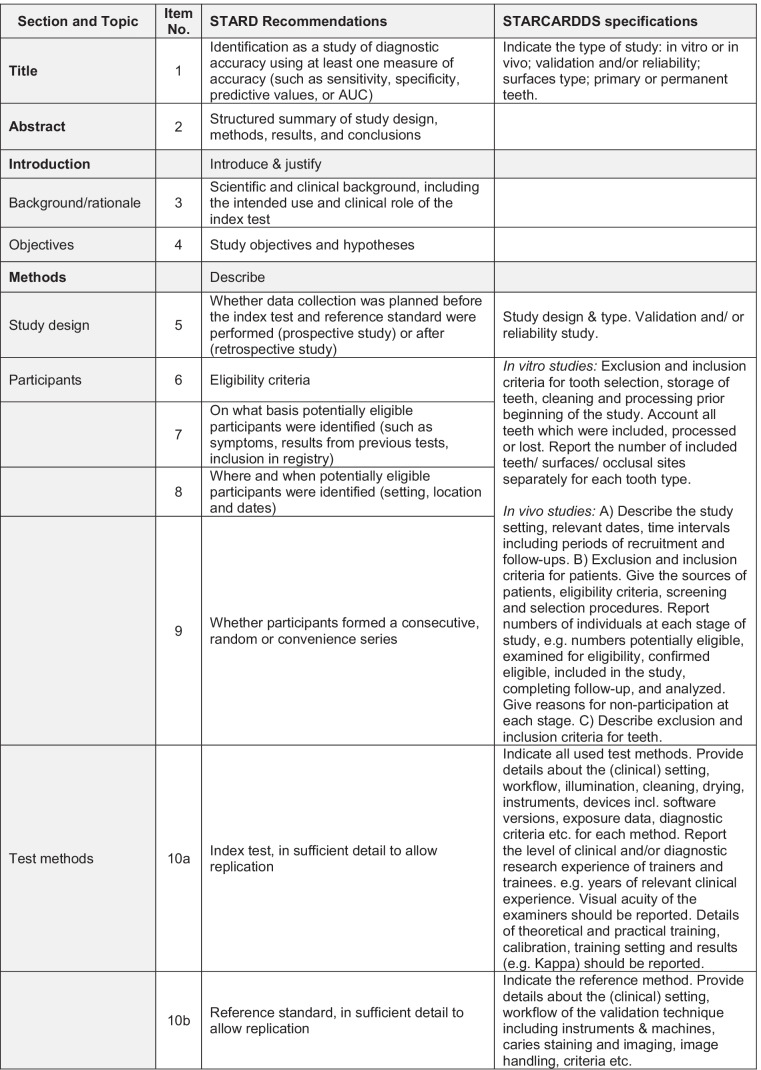

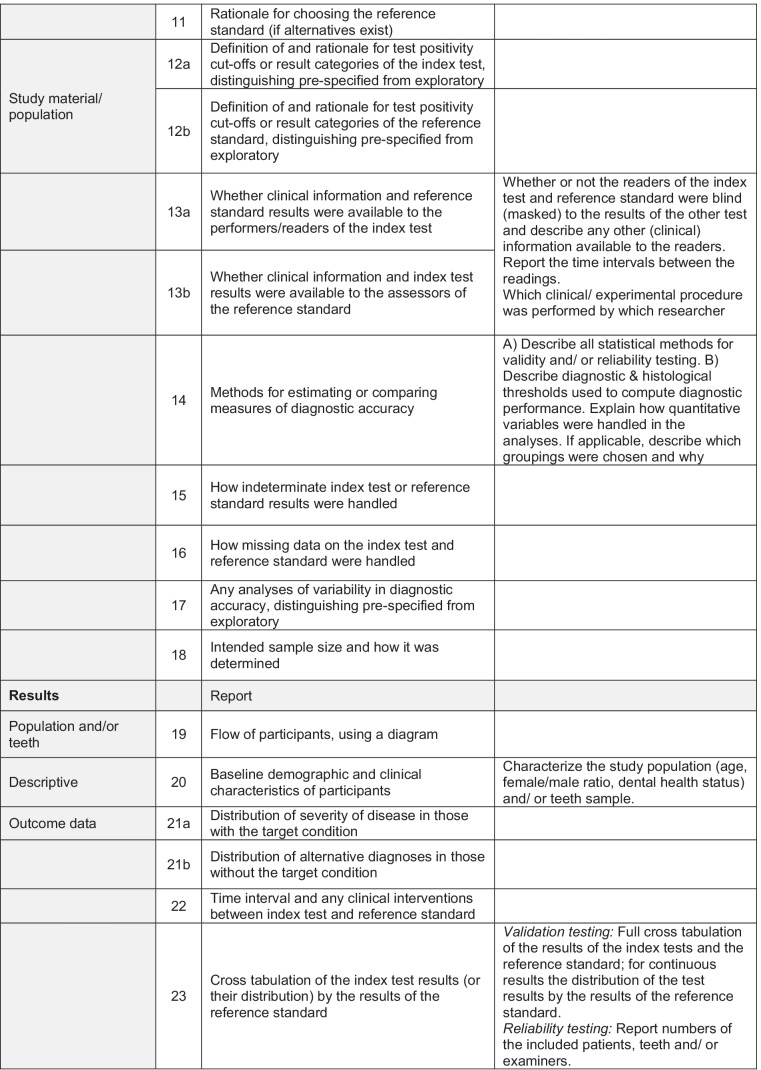

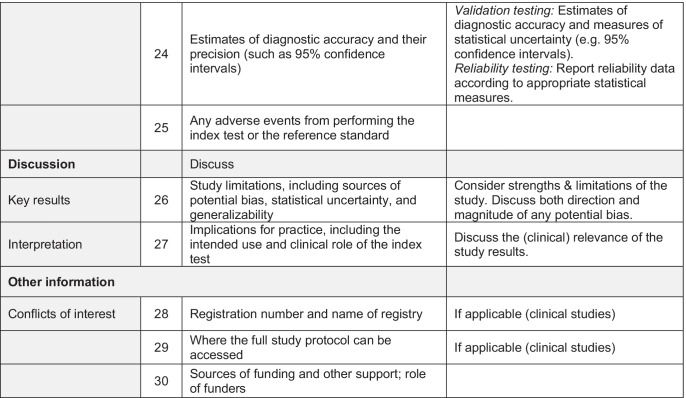
Recommendations and checklist for the STAndard Reporting of CAries Detection and Diagnostic Studies (STARCARDDS)

## Discussion

It was found that the STARD checklist needed refinement for caries detection and caries diagnosis studies in order to specifically target the research needs in cariology. Justifications are given below.

- *Item 1:* On top of common accuracy studies, for which sensitivity, specificity, positive or negative predictive value, or area under the ROC curve (AUC) are appropriate measures, in cariology, there also exist a number of studies addressing solely reliability. This type of studies aims at assessing objective criteria for reliability performance for new diagnostic methods [19] or, e.g., at assessing the influence of training and calibration [20]. Furthermore, because primary and permanent teeth may have different diagnostic thresholds, and because buccal smooth surface caries is easier to detect than interdental caries or occlusal caries, it is advisable to include information about the dentition (deciduous, permanent) and the assessed tooth surfaces of the study material. The title should describe which type of diagnostic study was conducted.
- *Item 5:* In addition to the STARD checklist, the type of study conducted should be specified (validation and/or reliability study, see item 1).
- *Items 6–9:* For in vitro caries diagnostic studies, full reporting is needed about the exclusion and inclusion criteria for tooth selection, storage of teeth, and any processing before execution of the study. This is especially important because it may represent a major source of bias. For instance, a caries diagnostic study may be carried out on teeth with an open apex, or teeth which have clearly been impacted and never been in function, teeth which would have a very low likelihood of having caries lesions. If this is obvious to the examiners, for instance because roots are left uncovered, then this information will likely lead to bias. Similar to other studies where pre-testing failures need to be reported [21, 22], also in caries detection studies, all teeth that were included, processed, or lost need to be fully accounted for. As different tooth types (e.g., premolars vs. molars) or tooth surfaces (e.g., smooth surface vs. occlusal surface) are not equally demanding for caries detection, the number of assessed teeth/surfaces should be given separately for each tooth type.

For in vivo studies, the STARD criteria are valid, but also require some further refinement. The study setting, relevant dates, time intervals, including periods of recruitment, and follow-ups need to be described. Furthermore, STARCARDDS elaborates on how to report the numbers of patients at each stage of the conducted study. Especially in patients with a mixed dentition, it seems to be important to exactly describe the reasons for inclusion and exclusion criteria of teeth. Special attention should be paid to how missing teeth are handled (missing due to caries vs. missing due to physiological exfoliation).

- *Item 10a:* In order to be able to compare the results of caries detection trials, the study settings should be standardized to the best of knowledge. It seems to be essential to particularly provide sufficient details about the clinical setting, the workflow, illumination conditions, eventual cleaning and/or drying of the tooth surfaces, and/or the supportive use of any instruments. If medical devices are used, their software version or exposure data should be provided. Because each diagnostic method in cariology has its own diagnostic criteria, they should be given in full detail. Furthermore, as clinical experience [20] and visual acuity are crucial factors in caries diagnostic studies [23, 24], it is indispensable to also report on the levels of clinical experience (years after graduation; specialist training) and on the visual acuity of the researchers.
- *Item 10b*: Histologic validation of caries varies between applied methods (e.g., [25, 26]). It seems crucial that, whenever some sort of histology serves as a reference standard, the workflow of the validation technique must be described in sufficient detail, including the use of instruments/machines, any staining of caries, the imaging process, the handling of the imaging, and the applied assessment criteria. Even though an absolute determination of the extension of the caries process is not possible (partly because of an ongoing discussion on the nature and level of tissue change that is relevant/critical in determining lesion progression stage), taking care of reporting all relevant details of the respective applied reference method helps to avoid study heterogeneity.
- *Item 11:* In in vivo caries trials, there is a problem how to validate healthy tooth surfaces or those tooth surfaces that do not need to be opened up. The latter case allows for immediate inspection of the depth of a lesion; the drilling of a lesion thus equals taking a biopsy in medicine. Though restorative caries treatment still represents a substantial part of everyday dental care provision, it is neither ethical nor helpful to operatively open up early stages of the caries process or even healthy surfaces. Therefore, many clinical diagnostic studies rely on imperfect reference standards such as radiography [27]. However, it seems to be an accepted method to directly inspect tooth surfaces after temporary tooth separation in order to validate early stages of caries or healthy surfaces [28]. While direct inspection of interdental caries after tooth separation has perfect specificity, the depth of more advanced carious lesions cannot be assessed by this procedure. It is therefore legitimate to discuss, for clinical caries studies, the use of a composite or hybrid reference standard [10] which on the one hand fully allows for assessment of healthy tooth surfaces and initial caries, and on the other hand for precise estimates of lesion depth. There have been concerns that composite reference standards may represent bias themselves because of their dependency of disease prevalence and their possibly underestimating or overestimating diagnostic accuracy [29]. However, using latent class models could render more precise estimates when using composite reference standards in in vivo caries diagnostic trials [30].
- *Items 13a and 13b:* The aspect of blinding is important also in caries diagnostic studies but often reported in insufficient detail. The steps to ensure blinding should be reported in a full and comprehensible way. Special attention should be paid to whether or not the readers of the index test and reference standard were blind (masked) to the results of the other test. Because in caries laboratory studies teeth are often assessed more than once, starting at sample selection and preparation, the time interval between the respective assessments needs to be reported [31]. Furthermore, the clinical and/or experimental procedures should be clearly attributable to the respective researchers who performed these steps.
- *Item 14:* Because in many caries diagnostic studies not only accuracy is assessed, but also reliability, all statistical methods for testing both validity and/or reliability have to be reported. With respect to the diagnostic and histological thresholds, a proper description is necessary. Because some diagnostic tests yield quantitative data, their statistical analysis must be reported as well. If grouping/clustering of data seems necessary (e.g., enamel caries vs. dentine caries), doing so has to be described and justified. Testing reliability between more than 2 examiners may require more sophisticated statistical methods (e.g., Fleiss’ Kappa [32], bootstrapping [33]) and these might be applied as well.
- *Item 20:* In in vitro experiments, the teeth used for the study have to be specified. Due to their characteristic appearance, it makes a diagnostic difference, if they have served in the oral cavity, or if they are newly erupted or impacted (see also items 6–9).
- *Item 23:* Next to full cross-tabulations, for reliability testing, the numbers of the included patients, teeth, and/or examiners need to be reported. In the case of multiple examiners, it can sometimes be observed that the diagnostic data are collapsed, or that “a consensus decision” was derived at. For the sake of transparency and confirmability, all data should be reported separately, or at least made public as an online supplemental content.
- *Item 24:* Next to testing of validation, where estimates of diagnostic accuracy and measures of statistical uncertainty (e.g., 95% confidence intervals) must be provided, reliability data also needs to be reported as well according to the appropriate statistical measures applied. This becomes especially relevant in trials with more than two observers.
- *Item 25:* Usually, no adverse effects can be noted in caries diagnostic trials. However, some methods use ionizing radiation or the application of dyes and thus could evoke adverse reactions. This item therefore needs to be addressed, too.

## Reporting in future diagnostic studies

There is evidence that insufficient reporting contributes to a higher risk of bias and to an increased heterogeneity between diagnostic accuracy studies [10]. Standardized reporting requirements are an important means to reduce heterogeneity among diagnostic accuracy studies. Most scientific journals try to standardize the way of reporting in their author guidelines in order to reduce heterogeneity of the reported data. STARCARDDS aims into the same direction but is specific with respect to caries diagnostic and detection studies, including reliability studies. STARCARDDS can, moreover, be used together with established guidelines such as the CONSORT statement [14] when reporting randomized clinical trials investigating caries detection and diagnostic methods. It is our hope that following the STARCARDDS checklist leads to a more complete reporting of study methodology and results, and will thus result in better comparability of future diagnostic studies in cariology.
